# Retraction: RPN2 promotes metastasis of hepatocellular carcinoma cell and inhibits autophagy via STAT3 and NF-κB pathways

**DOI:** 10.18632/aging.205555

**Published:** 2024-06-15

**Authors:** Linsheng Huang, Zhiyuan Jian, Yi Gao, Ping Zhou, Gan Zhang, Bin Jiang, Yi Lv

**Affiliations:** 1 National Local Joint Engineering Research Center for Precision Surgery and Regenerative Medicine, Xi’an, Shaanxi Province, China; 2 Department of Hepatopancreatobiliary Surgery, Taihe Hospital, Hubei University of Medicine, Shiyan, Hubei Province, China; 3 The First General Surgery Department of the Hospital Affiliated Guilin Medical University, Guilin, Guangxi Province, China; 4 Department of Hepatobiliary Surgery, The First Affiliated Hospital, Xi’an Jiaotong University, Xi’an, Shaanxi Province, China

**Keywords:** RPN2, HCC, EMT, MMP-9, STAT3

**This article has been retracted:** Aging has completed its investigation of this paper. We found multiple internal and external duplications and manipulations of the images. These include duplicating, rescaling and cropping of data published by other authors. We found that the following images were published in unrelated papers:  **Figures 3AB** and **5AB** [1]; **5E/F** [2]; **6A and 6AB** [3,4]; **6G** [5]; **6H** [6]; **7A** [7]; **9E** [8]. The first author admitted that he commissioned a biochemical company to do some experiments and later found that some of the data could not be reproduced. The authors replied that they “are unable to provide satisfactory data in relation to these figures and are unable to support the integrity of the research.” Consequently, all authors agreed that the article should be retracted.

The Administration of The First Affiliated Hospital, Xi’an Jiaotong University was notified about the retraction by Aging Journal.
